# Reward system and temporal pole contributions to affective evaluation during a first person shooter video game

**DOI:** 10.1186/1471-2202-12-66

**Published:** 2011-07-12

**Authors:** Krystyna A Mathiak, Martin Klasen, René Weber, Hermann Ackermann, Sukhwinder S Shergill, Klaus Mathiak

**Affiliations:** 1 Department of Child and Adolescent Psychiatry, Psychosomatics and Psychotherapy, RWTH Aachen University, Neuenhofer Weg 21, 52074 Aachen, Germany; 2 Department of Psychiatry, Psychotherapy and Psychosomatics, RWTH Aachen University, Pauwelsstr.30. 52074 Aachen, Germany; 3 King's College London, Institute of Psychiatry, Box P096, De Crespigny Park, SE5 8AF London, UK; 4 Hertie-Institute for Translation Brain Research, Graduate School of Neural & Behavioural Sciences, University of Tübingen, Hoppe-Seyler-Straße 3, 72076 Tübingen, Germany; 5 Jülich-Aachen Research Alliance (JARA) - Translational Brain Medicine, Germany; 6 Department of Communication, University of California, 4405 Social Sciences & Media Studies Building, Santa Barbara, CA 93106-4020, USA; 7 INM-1, Forschungszentrum Jülich GmbH, Wilhelm-Johnen-Straße, 52428 Jülich, Germany

## Abstract

**Background:**

Violent content in video games evokes many concerns but there is little research concerning its rewarding aspects. It was demonstrated that playing a video game leads to striatal dopamine release. It is unclear, however, which aspects of the game cause this reward system activation and if violent content contributes to it. We combined functional Magnetic Resonance Imaging (fMRI) with individual affect measures to address the neuronal correlates of violence in a video game.

**Results:**

Thirteen male German volunteers played a first-person shooter game (*Tactical Ops: Assault on Terror*) during fMRI measurement. We defined success as eliminating opponents, and failure as being eliminated themselves. Affect was measured directly before and after game play using the Positive and Negative Affect Schedule (PANAS). Failure and success events evoked increased activity in visual cortex but only failure decreased activity in orbitofrontal cortex and caudate nucleus. A negative correlation between negative affect and responses to failure was evident in the right temporal pole (rTP).

**Conclusions:**

The deactivation of the caudate nucleus during failure is in accordance with its role in reward-prediction error: it occurred whenever subject missed an expected reward (being eliminated rather than eliminating the opponent). We found no indication that violence events were directly rewarding for the players. We addressed subjective evaluations of affect change due to gameplay to study the reward system. Subjects reporting greater negative affect after playing the game had less rTP activity associated with failure. The rTP may therefore be involved in evaluating the failure events in a social context, to regulate the players' mood.

## Background

Playing interactive video games is an exciting aspect of the new media landscape that has experienced considerable growth during the last decade. Interactive gameplay has become a daily behavior, particularly for young people. In American children, game use increased from an average of 4 hours per week at the end of 1980s, to a current average of 13 hours per week [1,2]. At the same time, violent video games drew public attention. Particularly, there are controversies concerning first person shooter games. Content analyses of shooter games characterized the dominant (but not exclusive) narrative of those games as 'a human perpetrator engaging in repeated acts of justified violence involving weapons that results in bloodshed to the victim' [3].

Researchers have suggested that the violent content of games may desensitize players to real-world violence, serving to decrease their empathy and increase aggression [4-7]. Polman et al. [8] demonstrated that actively playing a video game leads to more short-term aggression than passively watching the same video, as expected from Bandura's social learning theory. Moreover, popular state-of-the-art video games are becoming more realistic and stimulate significantly more aggressive feelings and arousal over the course of play [9]. However, other researchers provided evidence that the effect sizes in meta-analysis might be a result of publication bias [10] and do not support the conclusion that violent video game playing leads to significant aggressive behavior. Similarly, the claim that playing violent video games (including first person shooters) lead to desensitization in the real world (e.g. towards depiction of violent behavior in real persons) has been challenged [11].

Despite the strong pressure from society, many games still contain a significant amount of violence. Indeed, violent media may enhance feelings of excitement, empowerment and satisfaction [12]. In contrast, Przybylski et al. [13] found that enjoyment and desire for future play were associated with the experience of autonomy and competence in game play, but not with the level of violence. Interestingly in this study, although players with high aggression trait preferred games with violent content, the violent content did not enhance their game enjoyment or immersion. Ravaja et al. [14] described that wounding and killing the opponents was accompanied by high-arousal with negative affect (anxiety) in players. This emotional response was lower in subjects with high psychoticism. So far, little research has focused on the rewarding aspects of video games or studied the reward system during playing.

Koepp et al. [15] demonstrated an involvement of the reward system in gameplay: striatal dopamine was released as a result of playing. The prominent role of the reward system in gameplay is also emphasized in a neuroimaging study by Hoeft et al. [16]. This study found activation in nucleus accumbens during a non-violent video game as compared to a control task. However, none of the above studies disentangled which events in the game contributed to the reward system activation. The striatal dopaminergic system is a part of a specialized network that is responsible for processing reward related cues. Other key structures include the orbitofrontal cortex and the midbrain dopamine neurons [17]. Those structures are involved in increasing the probability of behaviors leading to achieving a reward and avoiding a punishment, and consequently in directing our behavior [18]. Mathiak & Weber [19] found no influence of in-game violence events on midbrain reward structures as compared to the rest of the game play, which raises the question whether not only the respective events, but the gameplay as a whole is perceived as rewarding. In this case, content-specific influence on brain activation could be revealed by a comparison of rewarding with non-rewarding game phases. In a First Person Shooter (FPS) game, the moment of being killed (virtual dying) clearly indicates the unsuccessful ending of the round for the player and thus a failure in the gameplay. From a psychological perspective, this event of failing in a potentially rewarding situation is likely to correspond to a reward expectation or reward prediction error.

Reward expectation and reward prediction errors, that is the difference between expected outcome (reward or punishment) and the actual outcome, are thought to be critical for the dynamic adjustment in decision-making and reward-seeking behavior [20]. Reward prediction errors are encoded in structures including midbrain dopamine regions, the anterior and ventral portion of the striatum, the cingulate cortex, and the medial orbitofrontal cortex [21-24]. Phasic increases in activity are observed when outcomes are better than anticipated (a positive prediction error), and decreases are observed when outcomes are worse than expected (a negative prediction error; [25,26]). The involvement of the reward system during the game can encompass the anticipatory and appetitive phase of motivated behavior, which is engaged in learning which environmental stimuli or actions predict rewarding or punishing outcomes [15]. In a first person shooter game, the player cannot reliably predict if he will be able to survive the next fight, but he typically anticipates survival.

To study neural correlates underlying the complex self-motivated behavior engaged during natural unrestricted gameplay, it is possible to avoid traditional block or event-related paradigms, by utilizing a content analysis of experimenter-defined game events [19]. Neither traditional fMRI paradigms nor content analysis, however, directly control for inter-individual differences. Such control is particularly important in studies of a complex, multidimensional construct like the subjective experience of reward in a video game. A number of studies have successfully combined psychological inventories with fMRI to examine the neural correlates of psychological processes, especially highly subjective domains concerned with processing emotions and social interactions (e.g. [27,28], but see also [29] for methodological criticism).

Playing video games can have a strong impact on subjective and objective measures of player's affect, leading to increased aggressive emotions and physiological arousal [9,30,31]. Attaining a state of high arousal and excitement might serve as a motivation for persistent game play, analogous to that postulated in gamblers [32]. Although most of the studies have focused on negative affect, namely aggression, Coventry and Constable [33] recommended distinguishing between positive and negative affect when studying the maintenance of playing behavior. Indeed, a widely accepted model assumes that positive and negative affect dimensions are to large extent independent [34] and can be assessed using the Positive and Negative Affect Schedule (PANAS; [35]). Positive and negative affect have been differentially related to particular coping styles, life experiences and health concerns [36]. Watson [37] demonstrated that correlation between positive and negative affect is low and stable across different time frames when using the PANAS, supporting their relative independence. It is an alternative approach to the neuroscientifically well established concept, characterizing emotions according to valence and arousal, but may be more suitable to assess affective evaluation rather than emotions. The PANAS has been previously employed in functional neuroimaging studies - mostly to reveal the effectiveness of mood induction procedures [38]. Using verbal affect representation it may be particularly useful to assess subjective evaluation with slow changes.

Our aim was to determine the neuronal correlates of affective evaluation of game experience using functional Magnetic Resonance Imaging (fMRI). We hypothesized that game-related reward is associated with successful actions and the negative prediction errors are manifested when the player fails in a fight (while expecting to win). Moreover, we combined the objective measures of neuronal correlates - fMRI acquired during unrestricted video play - with quantitative reports of subjective affective evaluation - as measured with the PANAS. We applied the PANAS directly before and after the game to assess the change of positive and negative affect during the game play. Brain mapping should explore if brain activity to reward-related events was associated with the subjective evaluation. Specific hypothesis on neural mechanisms related to affect measures as assessed by the PANAS were not derived from the currently available data.

## Methods

### Subjects

Thirteen male German volunteers (age 18-26 years, mean 22.7 ± 2.0) were recruited by ads posted at the local university and in video game stores. We included right-handed subjects with at least 5 hours weekly video game experience (15.1 ± 9.0 hrs/week). We excluded individuals with contraindication against MR investigations, history of neurological, psychiatric, or ophthalmologic disorders. The study was approved by the local ethics committee. We obtained written informed consent from all participants involved in our study.

### Imaging Paradigm

The participants played a violent video game ("*Tactical Ops: Assault on Terror*," Infogrames Europe, Villeurbanne, France) during five (three of them only four) functional imaging session. In this shooter game derived from a widely-used game engine, the player experienced the action from the perspective of the virtual character that they control (first-person perspective), while other characters were controlled by the computer (bot mode); this procedure was chosen in order to avoid confounding variance due to varying game difficulties and event frequencies in the otherwise uncontrolled game environment. The game sound level was adjusted to comfortable listening levels. The players controlled the game using an MR-compatible trackball with five buttons; they had time to get acquainted with the controller before the fMRI experiment. Instructions were to create a motivational setting similar to that during online gaming.

During each 12 min session we recorded hemodynamic brain activity with triple-echo single-shot echo-planar imaging (EPI; repetition time TR = 2.25 s; echo times TE = 23, 40, and 62 ms; 64 × 48 matrix with 4 × 4 mm^2 ^resolution; 24 slices with 4 mm thickness plus 1 mm gap; 220 volumes) using a 3T MR scanner (Magnetom TRIO, Siemens, Erlangen, Germany). The video display of the game play with the audio track was recorded for content analysis and synchronization with the fMRI data was provided by recording the scanner pulses as second audio track. Different aspects of the data had been evaluated previously [19]. For functional coregistration, we acquired anatomical data from each participant before the functional sessions (T1-weighted 3 MPRAGE, 256 × 224 × 160 matrix with 1-mm isotropic voxels).

### Inventories

Subjects were asked to complete the Positive and Negative Affect Schedule (PANAS; [37]); for German version see [39]) directly before entering and after leaving the MR scanner. The scale contains 20 adjectives describing positive or negative emotions. Each item is rated on a 5-point scale ranging from 'very slightly or not at all' to 'extremely', leading to a total score of 10-50 points per scale. We compared scale for positive and negative affect from after the game with before start of the session (one-sample t-test).

### Content Analysis

The game structure of the first person shooter reveals one event of clear success and one for failure. These are virtual killing the opponents or being killed, respectively. The rater can readily identify both event types. The time-based content analysis was performed with two independent coders (male graduate students from the Annenberg School for Communication, University of Southern California) and one supervisor (R.W.). The recorded and digitized videos of the subjects' game play were analyzed frame by frame and event times were noted with 100-ms time resolution. A more complex content analysis with high time-resolution was used in these data [40,41]. The previous study [40] revealed that the basic narrative of FPS games like Tactical Ops develop around the killing and being-killed theme for most of the time. The killing events have a high-enough incidence to be analyzed in an fMRI event-related design. We therefore interpreted the virtual killing of an opponent, for which players receive points (reward), as a success event and being killed as failure. Moreover, our study expands this approach by relating the data to the subjective affect evaluation.

### fMRI Data Analysis

The reconstructed images underwent artifact reduction: construction of dynamic distortion maps from triple-echo EPI with alternating phase-encoding direction and subsequent matching of the three echoes [42], a combination of the three echoes weighted with TE*S_TE _based on expected contrast from the averaged signal decay [43]. Statistical parametric mapping was conducted following the standard SPM procedures with normalization into the Montreal Neurological Institute (MNI; [44]) template space of functional and anatomical data; smoothing (applying a spatial filter to increase signal-to-noise ratio and improve Gaussianity) with 12-mm full-width at half-maximum Gaussian kernel; general linear model constructed from the coding events convoluted with hemodynamic response function as independent variables; and random effect model for group analysis corrected for multiple testing across the entire brain volume. Movement parameters were obtained for realignment and assessed involuntary head motion of subjects. The head motion within each session was 0.30 ± 0.15 mm and thus comparable with other fMRI studies.

We assumed that success and failure elicited a neuronal response in circumscribed networks. The BOLD response was modeled by a generic hemodynamic response and achieved statistical maps for both types of events that were thresholded according to a voxel-wise corrected p < 0.05. Subsequently to test the impact of affective evaluation, we calculated the inter-subject regression models with the change of positive and negative affect measures from before to after the game as predictors for success and failure maps. Considering an inter-individual variability of networks subserving affective evaluation, we applied a cluster corrected threshold according to p < 0.05 (compare [43]).

In a subsequent exploratory analysis, we extracted individual contrast estimates from the relevant ROIs and determined the items of the PANAS questionnaire which were correlated (p < 0.05) with the localized reactivity; the number of above threshold items was considered for descriptive statistics. Calculations were conducted with SPM5 (Wellcome Department of Imaging Neuroscience, London, UK) and Matlab 7.1 (The Mathworks Inc., Natick, MA, USA).

## Results

All subjects had experience in playing first-person shooters and played the game successfully inside the fMRI scanner. They obtained an average of 82 success events (eliminating an opponent) and 26 failure events (being eliminated). Before the game, the participants scored on average 30.4 ± 4.0 on the positive and 13.0 ± 3.2 on the negative scale of PANAS. After game play, the average positive affect was 26.5 ± 5.1 and negative affect 11.8 ± 3.4. The descriptive statistics revealed a significant decrease of positive affect after the game (t(12) = 2.90, p = 0.013), but no change in negative affect across the group (t(12) = 1.14, p = 0.447).

**Figure 1:**
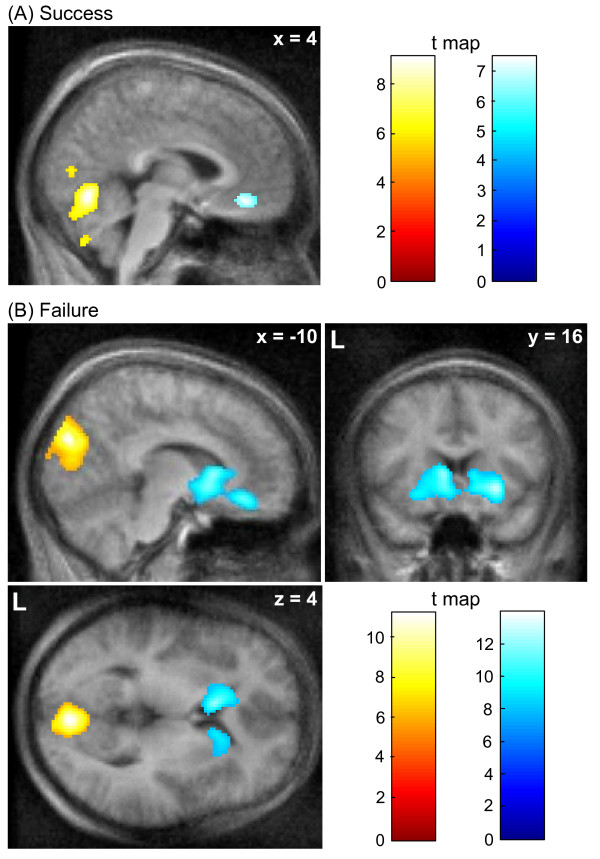
**Statistical maps on activation and deactivation during success (A) and failure events as compared to baseline (B; FWE corrected p < 0.05)**. Both success and failure were associated with increased visual or cerebellar activity. Success led to deactivation of the rACC. The caudate nucleus and orbitofrontal cortex showed deactivation in response to failure.

**Figure 2:**
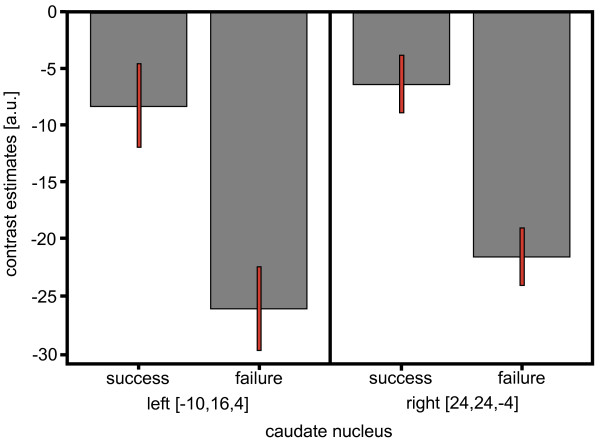
**Contrast estimates at the left and right caudate nucleus with 90% confidence interval**. The figure reflects the contrast between baseline and the success or failure events, respectively. Note that even after success event rather a trend to deactivation of the reward system can be observed.

Success events evoked increased activity in the cerebellum and decreased activity in the rostral ACC (family-wise error (FWE) corrected p < 0.05; Figure 1A; see the list of all observed clusters in Table 1). Failure events resulted in an increase of activation of visual areas and the cerebellum and a decrease in the orbitofrontal cortex and the caudate nucleus (FWE corrected p < 0.05; Figure 1B). Failure events affected more and larger clusters (Table 2) than success, but in the direct comparison the only significant clusters emerged in bilateral caudate nuclei (FWE corrected p < 0.05; not shown). The ROI analysis in bilateral caudate nuclei demonstrated that even after success events there was rather a trend for deactivation as compared to baseline gameplay than any sign of positive reward response (Figure 2).

**Table 1:** List of clusters (size > 100 voxels) activated and deactivated during success events (threshold according to FWE corrected p < 0.05)

ROI	cluster size	peak t-value	MNI coordinates			p-value
			x	y	z	
Activation in response to success events						

superior vermis	755	9.12	4	-72	-10	<0.001

inferior vermis	136	7.15	-2	-76	-42	0.003

Deactivation in response to success events						

rostral anterior cingulate gyrus	321	7.44	-8	38	-12	0.002

MNI - Montreal Neurological Institute; FWE - family-wise error

**Table 2:** List of clusters (size > 100 voxels) activated and deactivated during failure events (threshold according to FWE corrected p < 0.05)

ROI	cluster size	peak t-value	MNI coordinates			p-value
			x	y	z	
Activation in response to failure events						

visual dorsal pathway	1233	11.38	0	-84	26	<0.001

visual cortex R	108	8.86	16	-68	-4	<0.001

neocerebellar cortex L	392	8.6	-46	-58	-30	<0.001

Deactivation in response to failure events						

rostral anterior cingulate gyrus	321	7.44	-8	38	-12	0.002

caudate nucleus R	8031	14.05	24	24	-4	<0.001

caudate nucleus L	3035	11.98	-10	16	4	<0.001

intraparietal sulcus R	405	8.49	24	-60	58	<0.001

premotor R	678	7.27	30	-14	58	0.002

premotor L	328	7.01	-18	-2	58	0.004

intraparietal sulcus L	126	6.82	-22	-58	62	0.006

MNI - Montreal Neurological Institute; FWE - family-wise error; R - right; L - left

**Figure 3:**
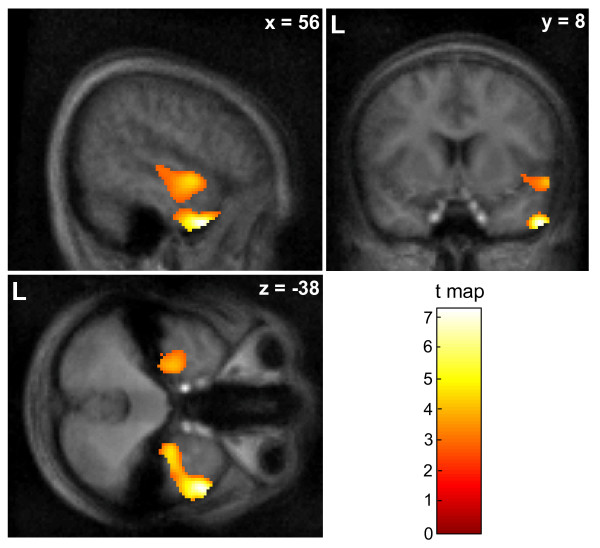
**Statistical maps on the behavioral prediction of lower individual responsiveness to failure events (cluster-size corrected p < 0.05)**. A negative association between failure response and negative affect were observed at the right temporal pole; other clusters comprised periaqueductal gray as well as orbitofrontal and premotor cortices (Table 3). No area predicted positive affect or higher negative affect.

The change of positive and negative affect from before to after the game served as a measure for affective evaluation. There was no reward system involvement evident during success events. Thus we focused on the correlation of affect measures with neural responses to failure. Negative affect correlated negatively with responses to failure in the right temporal pole (rTP) and to a lesser degree in the left temporal pole (lTP) and left orbitofrontal cortex (cluster-level corrected p < 0.05, Figure 3; Table 3). In other words, higher activity in TP during failure events was associated with reduced probability of a negative affective response to the game. No positive correlation with negative affect emerged and positive affect was not significantly associated with cortical activation.

**Table 3:** List of clusters (size > 100 voxels) involved in negative associations between failure response and negative affect (threshold for cluster size according to corrected p < 0.05)

ROI	cluster size	peak t-value	MNI coordinates			p-value
			x	y	z	
temporal pole R	12091	7.27	56	8	-38	<0.001

paracentral lobule	7873	5.84	-2	-34	44	<0.001

orbitofrontal cortex L	1825	4.76	-20	52	-22	0.004

MNI - Montreal Neurological Institute; R - right; L - left

The localized BOLD signal was extracted to identify items of the 20-item questionnaire which were correlated with the localized reactivity at a significance level of p < 0.05. In the rTP, activation was associated with reactivity to failure events for the following 8 items (expected 1 ± 0.97): excited (*aufgeregt*), anxious (*ängstlich*), hostile (*feindselig*), irritable (*reizbar*), nervous (*nervös*), timid (*furchtsam*), aggressive (*aggressiv*), and calm (*gelassen*); for all but the last item the association was negative. For the lTP, the following four items passed the significance level: excited, hostile, timid, and calm. For left and right caudate nucleus, only 2 and 3 items were above threshold which is compatible with a chance finding and suggests no systematic relation of the reward activity to failure.

## Discussion

We studied neural correlates of games experience during a first-person shooter game with fMRI. We found a relative deactivation of the caudate nucleus as well as the medial orbitofrontal cortex, as compared to the ongoing gameplay, when subjects failed in the game. This is in accordance with the reported role of those structures in reward-prediction error [24].

Negative reward prediction error is a decrease in activity observed when outcomes are more negative than expected or an anticipated reward is omitted [25,26]. Haruno and Kawato [45] demonstrated that caudate nucleus activation is correlated with reward-prediction-error during reward feedback. They argue that the caudate nucleus, like ventral striatum, is mainly engaged in the learning process involved in comparing actual and predicted rewards. In our study, deactivations occurred whenever the subject did not receive an expected reward, e.g. was eliminated instead of eliminating an opponent. A potential confound is a spill-over from motor structures in the dorsal striatum. However dorsal striatum did not yield a significant signal. Moreover the deactivation was apparent also during success events, which are linked to high motor activity.

In response to success events we found no activation of the structures responsible for reward processing. The majority of midbrain dopamine neurons show rather stereotyped, phasic activations following temporally unpredicted rewards, even if the delivery itself is to be predicted [46]. Although in our study the rate of success events was higher than of failures, there was a high level of temporal uncertainty included; therefore, we would expect reward system activation. Instead, we rather observed deactivation relative to baseline in the ROI analysis. This may be explained by rather tonic than phasic activation of the reward system present during the game as found by Koepp et al. [15]. Consequently this activation was not visible in the studied contrasts. Indeed the narrative of the game may lead to rewarding experience. In order to test this hypothesis, future studies could introduce a control game containing less rewarding phases and less stress on goal directed behavior. Przybylski et al. [13] suggested that reward in the game is not associated with the violent content, but factors such as experience of autonomy and competence in game play. In accordance with these behavioral findings, we found no activation of the reward system in response to violent events (even though they served as the direct aim in the game and players collected points for each time they eliminated an opponent). In our coding system, we considered killing an opponent as a rewarding event and did not differentiate how realistic the interaction was (compare [11]). Moreover, other factors such as motor activity may covary with different game events. This may explain the deactivations of premotor cortex associated with failure events (see Table 2) as sign of reduced activity after being killed. A more complex model of game enjoyment, that is not restricted to game violence and the direct aim of the game, may account for the broader spectrum of game experience (see [47]).

Subjects with larger right temporal pole (rTP) response to failure reported a decrease of negative affect after game play. TP activations have been frequently observed in simple emotional tasks, such as emotional face perception, as well as in complex emotional tasks, such as theory of mind, in particular with socially important narratives; moreover, the TP responded to tasks that require one to analyze other agent's emotions, intentions or beliefs (see [48]). Interestingly, although TP activations are frequently listed in fMRI results, their function is rarely discussed [49,50]. Olson et al. [48] suggested that the TP is involved in both social and emotional processes via binding complex, highly processed perceptual inputs to visceral emotional responses.

In our study, the rTP activation explained the individual differences in the increase of negative affect due to game playing: it was less active in response to failure in the game in those subjects who reported higher negative affect after the experiment. In the other subjects, rTP activation allowed them to evaluate the failure events in a broader cognitive and social frame of the game and protected them from affect and mood decreases. Indeed, patients with the atrophy of right but not left anterior temporal lobe present changes of mood including depression, apathy and irritability [51]. Moreover, neuroticism scores reflected TP involvement during the perception of negative emotions [52]. In a similar vein, Liu et al. [53] demonstrated that the TP was associated with an evaluation of wrong in-game decisions. We postulate that the TP can be involved in the evaluation of our own emotions in a broader social context, allowing to control our own affect in social situations, rather than assessing the intentions of others using theory of mind.

To further elucidate the function of the temporal pole and caudate nucleus in the individual appraisal of failure, we explored the prediction of single items by localized responses and found a profile of relevant adjectives. As compared to the rTP, the lTP showed less predictive power for the item and both caudate nuclei showed just a random association with the behavioral measures. The predicted adjectives (e.g. 'irritable' or 'hostile') were in agreement with the feeling of anger. In humans, uni- or bilateral anterior temporal lobe damage can lead to the Klüver-Bucy syndrome (see [48]). It was first described in monkeys, where it encompassed fear and anger and led to severe socio-emotional disorders [54]. Moreover, in a multivariate analysis of a community sample, Ferguson, Olson, Kutner & Warner [55] found the only form of aggression being predicted by video game violence was in response to anger (see Table 4 in [55]). This poorly specified feeling of anger may represent a central affect dimension in violent games and be controlled by right temporo-polar areas.

Unlike negative affect, positive affect change was not correlated with the change of brain responses. This is in accordance with the postulated model of independent positive and negative affect dimensions [34]. Moreover, it supports the claim of Coventry and Constable [33] who recommended measuring both positive and negative affect in gaming behavior. However, we chose a violent video game as an experimental paradigm and evaluated brain activation in response to violent events only. This may have biased us towards the experience of negative affect and also contribute to the lack of neural correlates to change in positive affect. It is less likely that the experience of being scanned rather than the game itself was responsible for the decrease in positive affect. Other researchers found no effect of scanning on the positive affect rating of PANAS (e.g. [38]). Moreover, violent games are known to evoke negative affective experiences, including aggressive feelings and thoughts [9].

Due to its location, the TP is an area susceptible to artifacts in fMRI due to the adjacent air-tissue transitions that lead to magnetic field inhomogeneities [56]. Therefore its activation can often be missing in neuroimaging data. We applied multiecho EPI sequence with alternating phase encoding polarity [42] and dephasing reduction [43] which allowed us to obtain robust signal from this region [57]. This improved technology lends additional credibility to the presented findings and may explain the lack of findings in previous fMRI studies.

The peak t-values at both temporal poles were low but the cluster extent was rather large. This finding suggests that the networks subserving the affective evaluation have a large variability across subjects or that the processing is distributed across extended structures. The compartmentalization at this level may be not as strict as in lower-level, motor or sensory areas (see [58]). This is in accordance with variability of other high-level functions such as language production [59]. Affective evaluation and its cognitive consequences may therefore be considered to emerge from distributed networks. Additional clusters implicated orbitofrontal and premotor structures as part of such extended network subserving the subjective evaluation. The reward areas and ACC failed to show significant associations with the affective measures and thus appear as distinct functional units.

Gaming behavior and reactivity of reward system are characterized by inter-individual variety. The Reinforcement Sensitivity Theory posits that a neurobiological system, the Behavioral Activation System, defines individual differences on the subject's sensitivity and reactivity to appetitive stimuli associated with mesocorticolimbic structures [60]. For instance, Bühler et al. [61] demonstrated the difference in processing of cigarette reward by occasional and dependent smokers: the former group demonstrated stronger reactivity of the mesocorticolimbic system for monetary than for cigarette reward, the latter responded equally to both reward types. Reuter et al. [62] reported a reduction of ventral striatal and ventromedial prefrontal activation in the pathological gamblers that was negatively correlated with gambling severity. Such variances of reactivity were not expected to specifically bias reward during gameplay or its relation to subjective experience. In larger study populations, the variables affecting reactivity in the reward system should be considered, which may prompt further insight into addictive video playing.

## Conclusion

We addressed for the first time the role of the reward system in the affective evaluation of playing a violent video game. We found no indication that violence events were directly rewarding for the players. The caudate nucleus was inhibited when the game outcome was worse than expected; this suggests that gamers continued playing to avoid failure experience. In contrast, the right temporal pole was involved in the affective evaluation of being repeatedly killed, allowing players to maintain a stable mood despite the failure events. The subjective measures of affect dimensions may help to disentangle different neural contributions to rewarding events during gameplay.

## Competing interests

The authors declare that they have no competing interests.

## Authors' contributions

KAM participated in data evaluation and interpretation and drafted the manuscript. MK helped to evaluate the data and draft the manuscript. RW helped to conceive and design the study and participated in data evaluation and drafting the manuscript. HA participated in conceiving the study, obtaining funds and drafting the manuscript. SSS was involved in data interpretation and drafting the manuscript. KM conceived and designed the study, acquired the funding, participated in and supervised collection of data, performed statistical analysis, and helped to draft the manuscript. All authors read and approved the final manuscript.
